# Physiological and Psychological Effects of Forest Therapy on Middle-Aged Males with High-Normal Blood Pressure

**DOI:** 10.3390/ijerph120302532

**Published:** 2015-02-25

**Authors:** Hiroko Ochiai, Harumi Ikei, Chorong Song, Maiko Kobayashi, Ako Takamatsu, Takashi Miura, Takahide Kagawa, Qing Li, Shigeyoshi Kumeda, Michiko Imai, Yoshifumi Miyazaki

**Affiliations:** 1 Department of Plastic and Reconstructive Surgery, National hospital organization Tokyo Medical Center, Higashigaoka 2-5-1, Meguro-ku, Tokyo 152-8902, Japan; E-Mail: ochiroko@gmail.com; 2 Center for Environment, Health and Field Sciences, Chiba University, Kashiwa-no-ha 6-2-1, Kashiwa, Chiba 277-0882, Japan; E-Mails: ikei.harumi@gmail.com (H.I.); crsong1028@gmail.com (C.S.); 3 Department of Hygiene and Public Health, Nippon Medical School, Sendagi 1-1-5, Bunkyo-ku, Tokyo 113-8602, Japan; E-Mails: mk831111@nms.ac.jp (M.K.); qing-li@nms.ac.jp (Q.L.); 4 Department of Plastic and Reconstructive Surgery, National Center for Child Health and Development, Okura 2-10-1, Setagaya-ku, Tokyo 157-8535, Japan; E-Mail: accodr@aol.com; 5 Agematsu Town Office Industry & Tourism Department, Agematsu 159-3, Kiso, Nagano 399-5601, Japan; E-Mail: syoukan@town.agematsu.nagano.jp; 6 Forestry and Forest Products Research Institute, 1 Matsunosato, Tsukuba City, Ibaraki Prefecture 305-8687, Japan; E-Mail: kagawa@ffpri.affrc.go.jp; 7 Nagano Prefectural Kiso Hospital, Kisomachi-fukushima 6613-4, Nagano 397-8555, Japan; E-Mail: kumeda@titan.ocn.ne.jp; 8 Le Verseau Inc., 3-19-4 Miyasaka, Setagaya-ku, Tokyo 156-0051, Japan; E-Mail: leverseau@mvb.biglobe.ne.jp

**Keywords:** forest therapy, high-normal blood pressure, adrenaline, cortisol, preventive medicine, Semantic Differential method, Profile of Mood State

## Abstract

Time spent walking and relaxing in a forest environment (“forest bathing” or “forest therapy”) has well demonstrated anti-stress effects in healthy adults, but benefits for ill or at-risk populations have not been reported. The present study assessed the physiological and psychological effects of forest therapy (relaxation and stress management activity in the forest) on middle-aged males with high-normal blood pressure. Blood pressure and several physiological and psychological indices of stress were measured the day before and approximately 2 h following forest therapy. Both pre- and post-treatment measures were conducted at the same time of day to avoid circadian influences. Systolic and diastolic blood pressure (BP), urinary adrenaline, and serum cortisol were all significantly lower than baseline following forest therapy (*p <* 0.05). Subjects reported feeling significantly more “relaxed” and “natural” according to the Semantic Differential (SD) method. Profile of Mood State (POMS) negative mood subscale scores for “tension-anxiety,” “confusion,” and “anger-hostility,” as well as the Total Mood Disturbance (TMD) score were significantly lower following forest therapy. These results highlight that forest is a promising treatment strategy to reduce blood pressure into the optimal range and possibly prevent progression to clinical hypertension in middle-aged males with high-normal blood pressure.

## 1. Introduction

While technology and modern city life offer unparalleled economic opportunities, conveniences, and comforts, urban environments are also stressful [1,2], which may contribute to chronic health problems. Many urban dwellers are thus looking for convenient methods of stress relief. Of these, the relaxing effects of natural environments are increasingly recognized as an effective counter to urban stress. The term “Shinrin-yoku” (taking in the atmosphere of the forest or literally “forest bathing”) was coined by the Japanese Ministry of Agriculture, Forestry and Fisheries to describe the positive effects of brief sojourns in natural environments to improve general health [3]. In later years, the term “Shinrin-yoku” developed into “Forest Therapy,” which uses the medically proven effects of walking and observing in a forest. Indeed, “Forest Therapy” is increasingly recognized as a relaxation and stress management activity with demonstrated clinical efficacy [4].

A variety of physiological indices show that humans are more relaxed in forested environments [3,4,5,6,7,8]. For example, a forest environment lowers blood pressure and pulse rate in humans [5,6,7]. Forest walking also suppresses sympathetic activity and increases parasympathetic activity [6,7] and reduces the levels of cortisol and cerebral blood flow in the prefrontal cortex [3]. It was also shown that a forest bathing trip can increase human natural killer (NK) cell activity and improve immunity in both males and females, and these effects were proved to last for at least 7 days [8]. In addition, psychological studies have demonstrated the benefits of forest environments on subjective measures of stress, cognitive function, and mood [5,6]. Park *et al.* reported the relaxation and stress management effects of forest environments by several questionnaire-based studies [9] as well as improved mood and cognitive function [10]. In psychological tests of young adult males, forest bathing significantly increased positive feeling scores and reduced negative feeling scores compared with urban stimuli [5,6,10].

However, previous studies have only investigated the physiological and psychological responses to forest bathing in healthy young adults, while such effects may be even more beneficial to middle-age subjects in the early stages of age-related diseases such as hypertension. Moreover, it is generally accepted that effects of treatment on blood pressure may vary between healthy normotensives and subjects with higher blood pressure, so studies on the latter population may be of greater clinical relevance. Therefore, the aim of this study was to measure the physiological and psychological effects of forest therapy on middle-aged males with high-normal blood pressure.

## 2. Materials and Methods

### 2.1. Participants

Nine Japanese males ranging in age from 40 to 72 years (56 ± 13.0; mean ± standard deviation) participated in this experiment. Potential participants who were taking medication for chronic conditions such as diabetes, hyperlipidemia, and hypertension were excluded. All participants had high-normal blood pressure (systolic 130–139 mmHg or diastolic 85–89 mmHg) as measured at Nagano Prefecture Kiso Hospital. Systolic blood pressure ranged from 124.5 to 137.5 mmHg (131.8 ± 4.1 mmHg) and diastolic blood pressure from 65.7 to 86.7 mmHg (77.3 ± 7.1 mmHg).

At the beginning of the experiment, subjects gathered in a waiting room at Nagano Prefecture Kiso Hospital and were fully informed about the study aims and procedures involved. After receiving a description of the experiment, the subjects all signed an agreement to participate. To control for the effect of alcohol, subjects did not consume alcohol during the entire study period. This study was approved by the Ethics Committee of Nagano Prefecture Kiso Hospital and the Center for Environment, Health and Field Sciences, Chiba University, Japan, on 19 August 2013 and performed according to the Declaration of Helsinki (1975, revised in 2008).

### 2.2. Experimental Sites

The forest therapy phase was conducted in Akasawa Shizen Kyuyourin (Akasawa Natural Recreation Forest), Agematsu, Nagano Prefecture (situated in central Japan) on 7 September 2013. Distance from the waiting room at Nagano Prefecture Kiso Hospital to the forest was 21.6 km, and it took 52 min to drive by car. The weather was cloudy, with a temperature of 21.5 °C (19.1 °C–25.0 °C) and humidity of 80.4% (62%–92%) on the day of forest therapy.

### 2.3. Physiological Indices

Systolic and diastolic blood pressure readings were obtained from the right arm using a portable digital sphygmomanometer (HEM-1020, Omron, Kyoto, Japan). Urine and blood samples were also obtained for the measurement of adrenaline, creatinine, and cortisol, respectively. All procedures were performed between 15:14 and 15:35 on the day before and a few hours after forest therapy to control for circadian effects. Participants were not allowed to talk each other during the measurement.

### 2.4. Psychological Indices

The Semantic Differential (SD) method, Profile of Mood State (POMS) subscale scores, and combined POMS Total Mood Disturbance (TMD) score were used to evaluate psychological responses to forest therapy. These questionnaires were completed by participants on the day before and soon after the experiment between 15:05 and 15:35. The SD method uses three pairs of adjectives anchoring seven-point scales: “comfortable to uncomfortable,” “relaxed to awakening,” and “natural to artificial” [11]. The POMS scores were determined for the following six subscales: “tension-anxiety (T-A),” “confusion (C),” “anger-hostility (A-H),” “depression (D),” “fatigue (F),” and “vigor (V).” A short form of the POMS with 30 questions was used to decrease the burden on the subjects [12]. The TMD score was calculated by combining T-A + C + A-H + D + F − V. A high TMD score indicates an unfavorable psychological state.

### 2.5. Experimental Design

**Figure 1:**
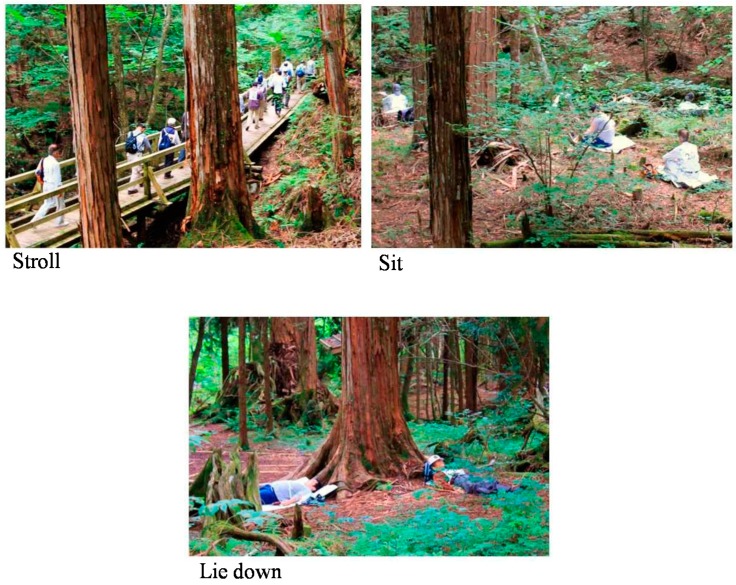
Images of the forest therapy experiment.

Participants spent the previous night in their respective homes. In the morning of the forest therapy day, participants gathered in the same meeting room at 9:00 a.m. and participated in the forest therapy program as a group with a guide. They were not allowed to communicate with each other during forest therapy, except during lunch time and designated rest periods, and they were not permitted to carry cell phones. Participants walked around their assigned area and then sat and lay on their backs in the forest on waterproof sheets laid on the ground; this program, comprising multiple actions, was performed for 4 h and 35 min (Table 1, Figure 1).

**Table 1:** Time schedules of and calorie consumption during various activities of forest therapy.

Time	Event	Calorie Consumption (Kcal/min)
10:30–11:08	Stroll (Forest)	0.92
11:09–11:20	Sit (Forest)	0
11:21–11:26	Stroll (Forest)	0.85
11:27–11:31	Deep breathing (Forest)	0.02
11:32–11.39	Stroll (Forest)	0.71
11:40–11:49	Lie down (Forest)	0
11:50–12:17	Stroll (Forest)	1.72
12:18–13:16	Lunch and rest (Resting room)	0.12
13:17–13:30	Stroll (Forest)	0.38
13:31–13:53	Ride on the “Forest train” (Forest)	0.04
13:54–13:58	Stroll (Forest)	0.64
13:59–14:16	Stroll (Indoor pavilion)	0.31
14:17–14:32	Stroll (Forest)	0.17
14:33–15:05	Rest (Resting room)	0.05

Energy expenditure for the activity was assessed (Lifecorder GS4; Suzuken Co., Ltd., Chiba, Japan). Tobacco and all drinks (except mineral water) were prohibited during forest therapy. They had the same lunch, which was made at lunch time from local ingredients. The subjects walked around their assigned areas and then sat and lay on their backs for 4 h and 45 min. Subjects then returned to a waiting room and completed the post-treatment measurements and questionnaires. These results were then compared with the data obtained on the day before.

### 2.6. Statistical Analysis

We used paired sample t-tests to compare physiological indices and the Wilcoxon signed-rank test to compare psychological test results before and after forest bathing. All statistical analyses were performed using SPSS 20.0 (IBM Corp., Armonk, NY, USA). Data are expressed as mean ± standard error (mean ± SE). For all cases, *p <* 0.05 (one sided) was considered statistically significant.

## 3. Results 

**Figure 2:**
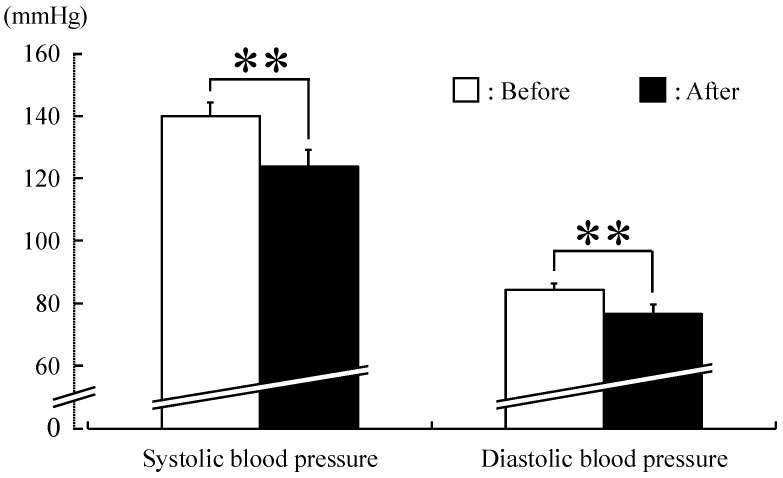
Effect of forest therapy on systolic and diastolic blood pressures in middle-aged males with high-normal blood pressure. *N =* 9, mean ± standard error. ** *p <* 0.01, paired *t*-test.

**Figure 3:**
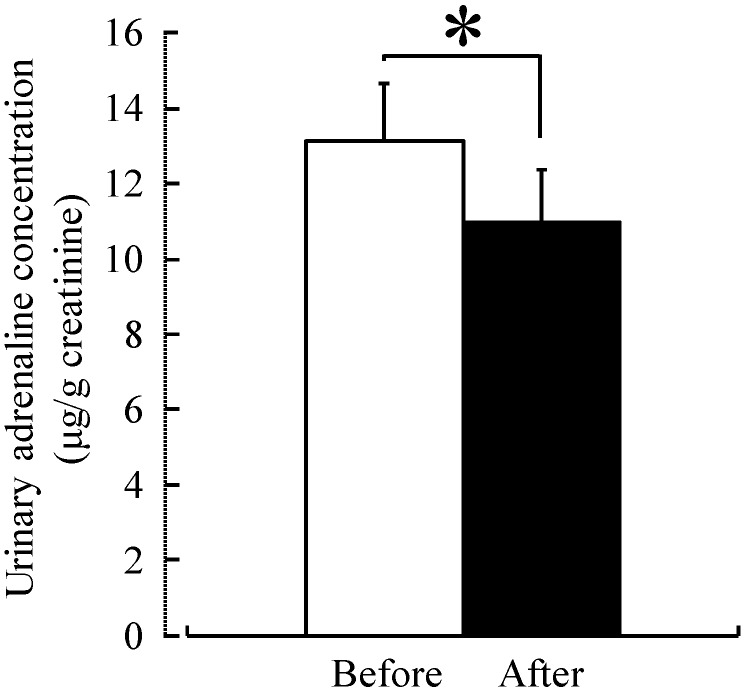
Effect of forest therapy on urinary adrenaline levels. *N =* 9, mean ± standard error. * *p <* 0.05, paired *t*-test.

**Figure 4:**
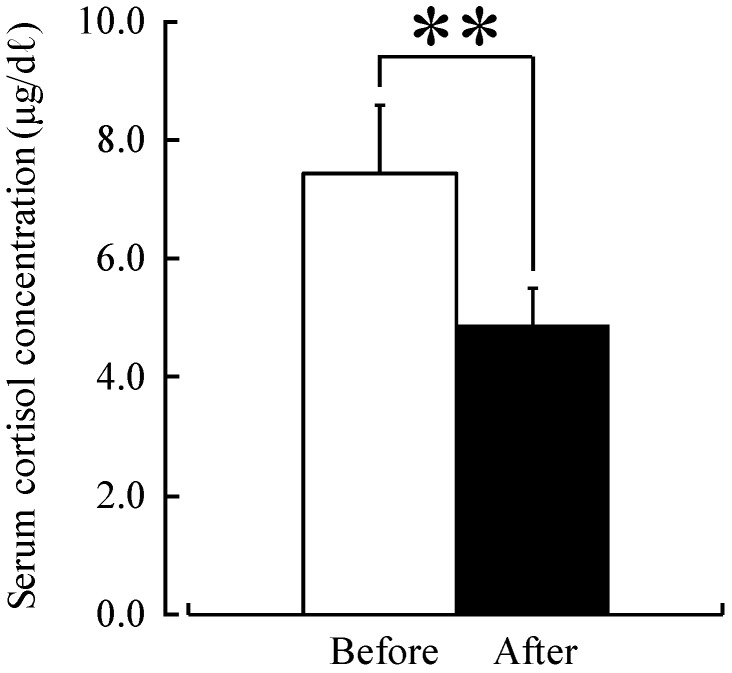
Effect of forest therapy on serum cortisol levels. *N =* 9, mean ± standard error. ** *p <* 0.01, paired *t*-test.

Both systolic and diastolic blood pressure were significantly lower after forest therapy (systolic blood pressure: before, 140.1 mmHg, after, 123.9 mmHg; diastolic blood pressure: before, 84.4 mmHg, after, 76.6 mmHg; *p <* 0.01) in middle-aged males with high-normal blood pressure (Figure 2). Similarly, both urinary adrenaline (with urinary creatinine correction) (before, 13.1 µg/g creatinine; after, 11.0 µg/g creatinine; *p <* 0.05) (Figure 3) and serum cortisol (before, 7.4 µg/dL; after, 4.9 µg/dL; *p <* 0.01) (Figure 4) were significantly lower after forest therapy.

**Figure 5:**
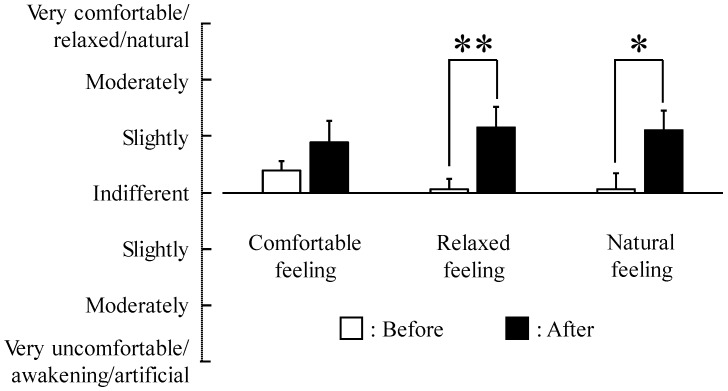
Semantic Differential (SD) method scores before and after forest therapy. Changes in the subjective feelings “comfortable,” “relaxed,” and “natural.” *N =* 9, mean ± standard error. ** *p <* 0.01, * *p <* 0.05, Wilcoxon signed-rank test.

**Figure 6:**
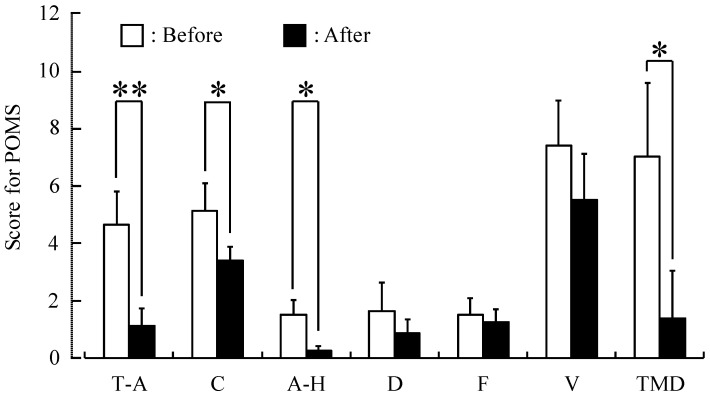
Subjective Profile of Mood State (POMS) scores before and after forest therapy. T-A, tension-anxiety; C, confusion; A-H, anger-hostility; D, depression; F, fatigue; V, vigor; TMD, Total Mood Disturbance. *N =* 8, mean ± standard error. ** *p <* 0.01, * *p <* 0.05, Wilcoxon signed-rank test.

Significantly higher semantic differential (SD) scores were observed for the adjectives “relaxed” (*p <* 0.01) and “natural” (*p <* 0.05) after forest therapy as compared with baseline (Figure 5). Finally, a significant elevation of mood was detected on the POMS test (Figure 6), with scores for the negative subscales “tension-anxiety” (*p <* 0.01) , “confusion,” and “anger-hostility” and the TMD significantly lower after forest therapy (*p <* 0.05). 

## 4. Discussion

This study assessed the physiological and psychological benefits of forest therapy on middle-aged Japanese men with high-normal blood pressure. Japanese guidelines for the management of hypertension (2014) [13] classify less than 140/90 mmHg as normal blood pressure and over 140/90 mmHg as high blood pressure. We enrolled only subjects diagnosed with “high-normal blood pressure” according to this definition. In general, the results were consistent with previous studies showing that forest therapy reduces multiple physiological and psychological indices of stress in healthy young adults [2,6,7]. Moreover, Li *et al.* reported that forest bathing significantly increased NK activity and decreased the concentration of adrenaline in urine, while a city tourist visit had no such effects [14]. However, the activities included in their forest therapy program were impossible to perform in an urban area, including meditation in front of a waterfall, embracing a tree, and the act of thinning of forest experiences. While the current study was preliminary in that we had no control group (*i.e*., both visiting the forest and visiting an urban area were not compared), we provide evidence for both physiological and psychological benefits in middle-aged patients at risk for hypertension. Regular forest therapy may thus prevent progression to clinical hypertension, a possibility warranting further investigation.

As blood pressure and many other physiological indices show a circadian rhythm, we paid special care to conduct pre- and post-treatment measurements at the same time (mid-afternoon) on successive days. Thus, circadian variation did not contribute to the changes reported. Forest therapy (including a leisurely walk and relaxation in a forest) reduced systolic blood pressure, urinary adrenaline, and serum cortisol. Blood pressure is under dual regulation by the sympathetic and parasympathetic nervous systems, with sympathetic activity increasing and parasympathetic activity reducing blood pressure [15]. Sympathetic activity can be determined by measuring the levels of urinary adrenaline and/or noradrenaline [16], and there are significant correlations between blood pressure and both urinary adrenaline and noradrenaline [15]. Moreover, many previous studies have shown that reducing stress decreases systemic cortisol [17] and sympathetic activity [17]. Thus, forest therapy may lower systolic and diastolic blood pressure of middle-aged males with high normal blood pressures by reducing sympathetic activity, consistent with previous studies on young healthy adults using multiple measures of stress response and autonomic activity, including cortisol and heart rate variability [3,5,6].

According to the SD and POMS questionnaires, participants felt more “comfortable,” “natural,” and “relaxed” after forest therapy. In addition, negative emotions were significantly reduced. Similarly, younger healthy subjects reported being significantly more comfortable and calm after walking in a forest compared to urban walks [18].

The risks of all cardiovascular diseases, strokes, myocardial infarction, chronic kidney disease, and associated risks of mortality increase in parallel with blood pressure above the optimum [19]. Thus, even patients with high-normal blood pressure benefit from methods that lower blood pressure. This patient group does not need antihypertensive agents; however, modification of lifestyles factors (such as a high sodium diet), weight loss, and exercise are recommended. The current study suggests that regular forest therapy is a convenient option to lower blood pressure into the optimal range and possibly to prevent progression to hypertension and associated complications.

From the viewpoint of public health, it is necessary to shift blood pressure downward in the entire population and not only in high-risk hypertensive patients [20]. Because forests occupy 67% of the land in Japan, they are easily accessible. Thus, forest therapy can be an effective and beneficial treatment for people of all ages and backgrounds. It is expected that broader application of forest therapy will improve the general health of the nation and reduce public medical expenses.

The present study provides evidence of physiological and psychological benefits of forest therapy for middle-aged males with high-normal blood pressure. However, the limitations of the present study include a lack of a control group performing similar activities in an urban environment. Furthermore, these results cannot yet be extrapolated to females or hypertensive adults. Studies examining health benefits in these groups are warranted in future study.

## 5. Conclusions 

Our study revealed that forest therapy elicited a significant: (1) decrease in systolic and diastolic blood pressure; (2) decrease in urinary adrenaline and serum cortisol levels; (3) increase in “relaxed” and “natural” feelings as assessed by the modified SD method; and (4) decrease in POMS negative subscales “tension-anxiety,” “confusion,” and “anger-hostility” as well as the TMD score in middle-aged males with high-normal blood pressure. Forest therapy may prevent progression to hypertension, thereby reducing associated risks of cardiovascular and renal diseases in this patient group.
